# Host Status of Cover Crops for the Management of *Pratylenchus Jaehni* on Coffee

**DOI:** 10.2478/jofnem-2022-0052

**Published:** 2022-11-17

**Authors:** Rosana Bessi, Mário Massayuki Inomoto

**Affiliations:** 1 Departamento de Fitopatologia e Nematologia, Escola Superior de Agricultura “Luiz de Queiroz”, Universidade de São Paulo, Piracicaba 13418-900, São Paulo, Brazil

**Keywords:** Brazil, *Crotalaria spectabilis*, green manure, *Helianthus annuus*, lesion nematode, sunflower, host-parasitic relatioship

## Abstract

The lesion nematode *Pratylenchus jaehni* occurs at low frequency in Brazilian coffee orchards but could provoke extensive root damage. Intercropping cover crops is a traditional practice in Brazilian coffee orchards, and the use of non-hosts of *P*. *jaehni* as cover crops may be a useful management method. In this work, 10 cover crops were tested concerning reproduction of *P*. *jaehni*. *Cajanus cajan*, *Canavalia ensiformis*, and *Mucuna deeringiana* are cover crops commonly used as intercropping in coffee orchards, but they must not be used in orchards infested with *P*. *jaehni*, because they are good hosts of this nematode. *Brachiaria ruziziensis*, *Crotalaria juncea*, *Dolichos lablab*, and *Pennisetum glaucum* were considered poor hosts. *Helianthus annuus* cv. Catissol and cv. Uruguai and *Crotalaria spectabilis* proved to be non-hosts to *P*. *jaehni*, and therefore, they are the cover crops recommended in coffee orchards infested with this nematode.

Intercropping cover crops in orchards of arabica and canephora coffee (*Coffea arabica* and *C*. *canephora*, respectively) is a traditional practice in many countries, due to the benefits of cover cropping on weed management, soil conservation, and reduction of chemical use in coffee farming (Opoku-Ameyaw *et al*., 2003; Santos *et al*., 2016). In Brazil, nitrogen-fixing plants, such as the mucunas (*Mucuna* spp.), pigeon pea (*Cajanus cajan*), jack bean (*Canavalia ensiformis*), and the crotalarias (*Crotalaria* spp.), are frequently used as cover crops for coffee because they improve the soil fertility by supplying organic nitrogen (Paulo *et al*., 2001; Bergo *et al*., 2006; Partelli *et al*., 2011). Furthermore, cover crops that are non-host to plant-parasitic nematodes may be used for the management of coffee-parasitic nematodes. For example, planting showy rattlebox (*Crotalaria spectabilis*) as cover crop between rows had been found to manage root-knot nematodes on coffee in Brazil (Jaehn, 1984).

In Brazil, the most prevalent nematodes in coffee plantations are *Meloidogyne exigua*, *Meloidogyne incognita*, and *Meloidogyne paranaensis* (Villain *et al*., 2018). Therefore, most of the efforts in nematode management in Brazil are dedicated to suppression of the root-knot nematodes. A survey of nematode infestation in coffee orchards of São Paulo state showed that *Pratylenchus brachyurus* occurred in 18.3% from 235 samples, *Pratylenchus jaehni* in 5.1%, and *Pratylenchus vulnus* in 0.4% (Kubo *et al*., 2004). In contrast to the situation prevailing with root-knot nematodes, the management of lesion nematodes in Brazilian coffee orchards is often being ignored.

*Pratylenhus jaehni* was formerly classified as *Pratylenchus coffeae* with three distinct populations: C1, C2, and K5 (Duncan *et al*., 1999; Inserra *et al*., 2002). In pot experiments, both C1 and C2 reproduced on Rangpur lime (*Citrus limonia*) but not on arabica coffee (Wilcken *et al*., 2008; Bonfim *et al*., 2011; Oliveira *et al*., 2011), while the population K5 reproduces on coffee (Silva and Inomoto, 2002). Inomoto *et al*. (2004) reported that the population K5 is as aggressive as *M. incognita* to the arabica coffee plants. Considering the extensive damage caused by population K5 of *P*. *jaehni* in the roots of arabica coffee and some cultivars of canephora coffee (Tomazini *et al*., 2005; Inomoto *et al*., 2008), and the absence of nematicides (biological and synthetic) for *P*. *jaehni*, there is a need to find a cover crop that is effective in managing *P. jaehni*. The objective of this project is to evaluate the host status of 10 tropical cover crops commonly used in Brazil to suppress *P. jaehni*.

## Materials and methods

Two glasshouse experiments were conducted during the summer of 2010 to 2011 (Experiment 1) and the fall of 2011 (Experiment 2) at Escola Superior de Agricultura “Luiz de Queiroz,” in Piracicaba (22^o^42×S; 47^o^38×W; 546 m of altitude), São Paulo, Brazil. During the experiments, average daily maximum and minimum temperatures in the glasshouse were 28.9 ± 2.5°C and 20.0 ± 1.6°C in Experiment 1, and 31.4 ± 2.8°C and 15.2 ± 3.5°C in Experiment 2. In Experiment 1, the 10 cover crops tested were: dwarf mucuna (*Mucuna deeringiana*), hyacinth bean (*Dolichos lablab*), jack bean (*Canavalia ensiformis*), Kennedy ruzi grass (*Brachiaria ruziziensis*), pearl millet cv. ADR-300 (*Pennisetum glaucum*), pigeon pea cv. Iapar-43 (*Cajanus cajan*), showy rattlebox (*Crotalaria spectabilis*), sunflower cv. Catissol and cv. Uruguai (*Helianthus annuus*), and sunn hemp cv. IAC-KR-1 (*Crotalaria juncea*). In Experiment 2, cover crops that allowed lower nematode reproduction in Experiment 1 were reevaluated (Kennedy ruzi grass, pearl millet cv. ADR-300, showy rattlebox, sunflower cv. Catissol, and cv. Uruguai, sunn hemp cv. IAC-KR-1). Grain sorghum cv. Sara (*Sorghum bicolor*) was included in both experiments as positive control, as it is a good host of *P*. *jaehni* (Silva and Inomoto, 2002). Coffee plants were not included in the experiments as the nematode population builds up slowly on coffee (Silva and Inomoto, 2002), and for this reason, coffee plants were considered unsuitable for comparison with cover crops.

## Seedling preparation and nematode inoculum

The substrate used in experiments was sandy clay loam soil (75.2% sand, 3.4% silt, 21.4% clay, 1.3% organic matter, pH 6.3) treated with steam heat (121°C for 2 hr). Seeds of the cover crops were sowed directly in the pots (12-cm tall and 7.3-cm diam. plastic pots containing 480 cm^3^ of soil). At 7 d after germination, the seedlings were thinned to three plants per pot in Experiment 1, and two plants per pot in Experiment 2, as these numbers were found adequate, considering the size of the pots.

Nematode inoculum of *P. jaehni* was the same as that used in the studies of Silva and Inomoto (2002), and Oliveira *et al*. (2011). They were obtained from roots of arabica coffee collected in the municipality of Marília, São Paulo state, in 1998. According to Oliveira *et al*. (2011), this isolate showed high homology level on D2–D3 expansion fragments (99% and 98%, respectively) with an isolate of *P*. *jaehni* deposited in the GenBank. The nematode was maintained in the laboratory on alfalfa callus (Riedel *et al*., 1973), and in glasshouse on roots of grain sorghum cv. Sara. Inoculum of *P*. *jaehni* was extracted from sorghum roots by a modified Baermann method (Hooper, 1986), and prepared to Pi of 200 juveniles and adults per pot. The *P. jaheni* inoculum (2 mL) was distributed into two holes per pot made in the soil, close to either side of the seedlings. After the nematode inoculation, the seedlings were maintained in a shaded place for 2 d to avoid heat stress on the nematode and then were transferred to a glasshouse, where they were maintained until evaluation.

### Experimental design

Both experiments were established in a completely randomized design with 11 treatments and six replicates (Experiment 1), and seven treatments and six replicates (Experiment 2).

### Evaluation

Final density of *P*. *jaehni* on the cover crops was assessed 75 d after inoculation in Experiment 1, and 90 d after inoculation in Experiment 2. Testing periods were based on the cycle of pearl millet, which is the tested plant with a shorter life cycle (100 d approximately). Pots were immersed in a bucket containing 4 L of tap water in order to separate the roots from the soil. Roots were washed with tap water, dried on absorbent paper, cut in 1-cm pieces, and weighted. A subsample of 10 g of each replicate was processed by blender followed by centrifugal-flotation (Coolen and D’Herde, 1972), using a centrifuge with four 126-mL tubes (10-cm high and 4-cm diam.) at 580 *g*. Pf was estimated by counting juveniles and adults recovered from the roots. The variables used for comparison were the Rf, i.e., the ratio between the Pf and the Pi, and nematode per gram of fresh root.

### Statistical analysis

All data collected were subjected to one-way analysis using SAS statistical software (SAS Institute, 2003). Means were separated by Tukey’s Honestly Significant Difference Test (*P* = 0.05) wherever appropriate. The Rf of the cover crops was compared with the Rf of sorghum, which is a good host of *P*. *jaehni* (Silva and Inomoto, 2002). Plants with Rf > 1 and not differing statistically from sorghum were considered good hosts of *P*. *jaehni*; plants with 1 < Rf < Rf of sorghum were considered poor hosts; and plants with Rf < 1 were considered non-hosts.

## Results and Discussion

In Experiment 1, jack bean, dwarf mucuna, and dwarf pigeon pea cv. Iapar-43 were rated as good hosts of *P*. *jaehni*; hyacinth bean and Kennedy ruzi grass were poor hosts; and sunflower cv. Uruguai, sunflower cv. Catissol, sunn hemp cv. IAC-KR-1, pearl millet cv. ADR-300, and showy rattlebox were non-hosts (Table 1). Results from Experiment 2 were similar to Experiment 1, where Kennedy ruzi grass was a poor host, while sunflower cv. Uruguai, sunflower cv. Catissol, and showy rattlebox were non-hosts. Although sunn hemp cv. IAC-KR-1 and pearl millet cv. ADR-300 were rated as non-hosts in Experiment 1, they allowed a low reproduction rate of *P*. *jaehni*, and therefore should be considered as poor hosts.

**Table 1:** Reproduction factors (Rf) of *Pratylenchus jaehni* and nematodes per fresh root mass (Nem/g) of cover crops at 75 d (Experiment 1) and 90 d after nematode inoculation (Experiment 2).

Cover crop	Experiment 1		Experiment 2	
	Rf	Nem/g	Rf	Nem/g
Grain sorghum cv. Sara	6.68^a,1^	28.78^a,b^	5.14^a^	12.83^a^
Jack bean	5.37^a^	46.06^a^	-	-
Dwarf mucuna	3.10^a,b^	8.61^b,c^	-	-
Pigeon pea cv. Iapar-43	2.08^a,b,c^	6.50^b,c,d^	-	-
Hyacinth bean	1.65^b,c,d^	6.89^b,c,d^	-	-
Kennedy ruzi grass	1.56^b,c,d,e^	9.97^b,c^	2.06^b^	12.94^a^
Sunflower cv. Uruguai	0.49^c,d,e^	4.08^c,d,e^	0.30^c,d^	2.17^b^
Sunn hemp cv. IAC-KR-1	0.36^d,e^	1.92^c,d,e^	1.35^b,c^	5.72^a,b^
Pearl millet cv. ADR-300	0.21^e^	1.42^d,e^	1.69^b^	14.17^a^
Sunflower cv. Catissol	0.12^e^	1.17^d,e^	0.15^d^	2.24^b,c^
Showy rattlebox	0.01^e^	0.08^e^	0.00^d^	0.00^c^

1 Means (*n* = 6) followed by the same letter(s) in a column are not different according to Tukey’s test (*P* = 0.05).

Intercropping of jack bean, dwarf mucuna, hyacinth bean, and pearl millet was suggested to provide weed management in coffee orchards (Partelli *et al*., 2010; Martins *et al*., 2015; Santos *et al*., 2016). Jack bean, dwarf mucuna, and pigeon pea are leguminous cover crops that can fix nitrogen, and upon termination of these cover crops, their residues may supply part of the nitrogen needs for coffee (Partelli *et al*., 2011). Legumes such as showy rattlebox, pigeon pea, and hyacinth bean had been demonstrated to increase coffee productivity when intercropping as ground cover with coffee (Guimarães *et al*., 2016). However, cover crops planted too close to the coffee plants may provoke coffee yield reduction, due to competition for water, light, and nutrients (Paulo *et al*., 2001). The current study showed that another detrimental effect of cover crops may be the increase of population density of *P. jaehni*.

The current study demonstrated that jack bean, dwarf mucuna, and pigeon pea were good hosts of *P*. *jaehni* (population K5). Therefore, they should not be used as cover crops in coffee orchards infested with this nematode. This is the first report of hyacinth bean and Kennedy ruzi grass as poor hosts of *P*. *jaehni* (population K5), as they only allow a smaller increase in population density of the nematode than grain sorghum. Sunn hemp and pearl millet were non-hosts of *P*. *jaehni* (Rf < 1) in Experiment 1 but were poor hosts (Rf > 1) in Experiment 2. This result was different from a previous study where an unknown cultivar of pearl millet was identified as a good host (Rf = 3.50 after 70 d, not differing from Rf = 6.27 of sorghum cv. IPA-7301011) of the population K5 of *P*. *jaehni* (Silva and Inomoto, 2002). Inconsistency of Rf values for sunn hemp and pearl millet on *P. jaehni* suggested that these cover crops should not be recommended for intercropping with coffee in a *P. jaehni* infested field. When the host suitability of the same cover crops was assessed for *P. brachyurus*, both Kennedy ruzi grass and pearl millet cv. ADR-300 were considered poor hosts (Inomoto and Asmus, 2010), whereas sunn hemp (Machado *et al*., 2007) and hyacinth bean (unpubl. data) were rated as good hosts.

Sunflower is another cover crop used in coffee orchards in Brazil (Wutke *et al*., 2014; Espindula *et al*., 2015). Including the current study, three sunflower cultivars (cv. Uruguai, cv. Catissol, and cv. Morgan-734) are now confirmed to decrease the population density of *P*. *jaehni* (Silva and Inomoto, 2002). However, intercropping is recommended only in the first 3 yr of coffee crop to avoid competition with coffee plants (Espindula *et al*., 2015). Notwithstanding, Asmus *et al*. (2005) reported that sunflower cv. Uruguai was a good host to *M. incognita*. Similarly, Dias-Arieira *et al*. (2008) found that eight sunflower cultivars, including cv. Catissol, were susceptible to *M. incognita*. Conversely, the same authors found four resistant cultivars of sunflower to *M. paranaensis*, another important plant-parasitic nematode of coffee plants. As for *P. brachyurus*, Dias *et al*. (2016) identified several sunflower genotypes resistant to this species but not to *M. incognita*.

Showy rattlebox was never tested for its host status on *P*. *jaehni*. This cover crop has been largely used in double-crop with soybean to manage *P*. *brachyurus*, mainly in the Central-West region of Brazil, with an estimated 4 million ha sowed in 2018 (Souza and Inomoto, 2019). It is considered as a non-host to *P. brachyurus* (Machado *et al*., 2007). Previous reports also demonstrated its resistance to *M. incognita* (Linde, 1956), and *M. exigua* (Almeida and Campos, 1991). The suitability of this cover crop for *M. paranaensis* is unknown.

In conclusion, showy rattlebox and sunflower may be used as cover crops for intercropping with coffee in orchards infested by the population K5 of *P*. *jaehni* due to their non-host status to this plant-parasitic nematode on coffee. Whereas hyacinth bean and Kennedy ruzi grass are rated as poor hosts of *P. jaehni*, jack bean, dwarf mucuna, and pigeon pea should not be intercropped with coffee in a population K5 *P. jaehni* infested field due to their high susceptibility to this population of nematode.

## References

[j_jofnem-2022-0052_ref_001] Almeida V. F., Campos V. P. (1991). Reprodutividade de Meloidogyne exigua em plantas antagonistas e em culturas de interesse econômico. Nematologia Brasileira.

[j_jofnem-2022-0052_ref_002] Asmus G. L., Inomoto M. M., Sazaki C. S. S., Ferraz M. A. (2005). Reação de algumas culturas utilizadas no sistema plantio direto a Meloidogyne incognita. Nematologia Brasileira.

[j_jofnem-2022-0052_ref_003] Bergo C. L., Pacheco E. P., Mendonça H. A., Marinho J. T. S. (2006). Avaliação de espécies leguminosas na formação de cafezais no segmento da agricultura familiar no Acre. Acta Amazonica.

[j_jofnem-2022-0052_ref_004] Bonfim Jr., Oliveira C. M. G., Inomoto M. M. (2011). Reação de porta-enxertos cítricos à população K5 de Pratylenchus jaehni. Tropical Plant Pathology.

[j_jofnem-2022-0052_ref_005] Coolen W. A., D’Herde C. J. (1972). A method for the quantitative extraction of nematodes from plant tissue.

[j_jofnem-2022-0052_ref_006] Dias W. P., Moraes L. A. C., Carvalho C. G. P., Oliveira M. C. N., Orsini I. P., Leite R. M. V. B. C. (2016). Resistance to Meloidogyne incognita Meloidogyne javanica and Pratylenchus brachyurus in sunflower cultivars adapted to the tropical region of Brazil. Tropical Plant Pathology.

[j_jofnem-2022-0052_ref_007] Dias-Arieira C. R., Santana S. M., Silva M. L., Furlanetto C., Ribeiro R. C. F., Lopes E. A. (2008). Reação de cultivares de mamona (Ricinus communis L.) e girasol (Helianthus annuus L.) a Meloidogyne javanica M. incognita e M. paranaensis. Nematologia Brasileira.

[j_jofnem-2022-0052_ref_008] Duncan L. W., Inserra R. N., Thomas S. K., Dunn D., Mustika I., Frisse L. M., Mendes M. L., Morris K., Kaplan D. T. (1999). Molecular and morphological analyses of isolates of Pratylenchus coffeae and closely related species. Nematropica.

[j_jofnem-2022-0052_ref_009] Espindula M. C., Marcolan A. L., Costa R. S. C., Ramalho A. R., Docleciano J. M., Santos J. C. F., L. Marcolan A., C. Espindula M., na Amazônia Café (2015). Implantação da lavoura.

[j_jofnem-2022-0052_ref_010] Guimarães G. P., Lima P. C., Moura W. M., Valente R. F., Guarçoni A., Mendonça E. S. (2016). Productivity of coffee and legumes intercropped under different sun exposure face. Australian Journal of Crop Science.

[j_jofnem-2022-0052_ref_011] Hooper D. J., F. Southey J. (1986). Laboratory methods for work with plant and soil nematodes.

[j_jofnem-2022-0052_ref_012] Inomoto M. M., Asmus G. L. (2010). Host status of graminaceous cover crops for Pratylenchus brachyurus. Plant Disease.

[j_jofnem-2022-0052_ref_013] Inomoto M. M., Beluti D. B., Siqueira K. M. S., Kubo R. K. (2004). Efeito de Pratylenchus coffeae e Meloidogyne incognita no crescimento de cafeeiro ‘Catuaí Vermelho’. Nematologia Brasileira.

[j_jofnem-2022-0052_ref_014] Inomoto M. M., Kubo R. K., Silva R. A., Oliveira C. M. G., Tomazini M. D., Mazzafera P. (2008). Pathogenicity of two Pratylenchus coffeae populations from Brazil on coffee plants. Nematology.

[j_jofnem-2022-0052_ref_015] Inserra R. N., Duncan L. W., Trocoli A., dos Santos J. M., Kaplan D., Vovlas N. (2002). Pratylenchus jaehni sp. n. from citrus in Brazil and its relationships with P coffeae and P loosi (Nematoda: Pratylenchidae). Nematology.

[j_jofnem-2022-0052_ref_016] Jaehn A. (1984). Recuperação de lavoura cafeeira recepada, com utilização de Crotalaria spectabilis, torta de mamona e nematicidas, em área infestada por Meloidogyne incognita. Nematologia Brasileira.

[j_jofnem-2022-0052_ref_017] Kubo R. K., Oliveira C. M. G., Antedomênico S. R., Monteiro A. R., Ferraz L. C. C. B., Inomoto M. M. (2004). Ocorrência de nematoides do gênero Pratylenchus em cafezais do estado de São Paulo. Nematologia Brasileira.

[j_jofnem-2022-0052_ref_018] Linde W. J. Van der. (1956). The Meloidogyne problem in South Africa. Nematologica.

[j_jofnem-2022-0052_ref_019] Machado A. C. Z., Motta L. C. C., Siqueira K. M. S., Ferraz L. C. C. B., Inomoto M. M. (2007). Host status of green manures for two isolates of Pratylenchus brachyurus in Brazil. Nematology.

[j_jofnem-2022-0052_ref_020] Martins B. H., Araujo-Junior C. F., Miyazawa M., Vieira K. M., Milori D. M. B. P. (2015). Soil organic matter quality and weed diversity in coffee plantation area submitted to weed control and cover crops management. Soil and Tillage Research.

[j_jofnem-2022-0052_ref_021] Oliveira C. M. G., Bessi R., Harakava R., Machado A. C. Z., Kubo R. K. (2011). Técnicas moleculares e microscopia eletrônica de varredura no esclarecimento da posição taxonômica da população K5 de Pratylenchus sp. Nematologia Brasileira.

[j_jofnem-2022-0052_ref_022] Opoku-Ameyaw K., Oppong F. K., Ofori-Frimpong K., Amoah F. M., Osei-Bonsu K. (2003). Intercropping robusta coffee with some edible crops in Ghana: Agronomic performance and economic returns. Ghana Journal of Agricultural Science.

[j_jofnem-2022-0052_ref_023] Partelli F. L., Vieira H. D., Ferreira E. P. B., Viana A. P., Espíndola J. A. A., Urquiaga S., Boddey R. M. (2011). Biological dinitrogen fixation and nutrient cycling in cover crops and their effect in organic Conilon coffee. Semina: Ciências Agrárias.

[j_jofnem-2022-0052_ref_024] Partelli F. L., Vieira H. D., Freitas S. P., Espíndola J. A. A. (2010). Aspectos fitossociológicos e manejo de plantas espontâneas utilizando espécies de cobertura em cafeeiro Conilon orgânico. Semina: Ciências Agrárias.

[j_jofnem-2022-0052_ref_025] Paulo R. M., Berton R. S., Cavichioli J. C., Bulisani E. A., Kasai F. S. (2001). Produtividade do café Apoatã em consórcio com leguminosas na região da Alta Paulista. Bragantia.

[j_jofnem-2022-0052_ref_026] Riedel R. M., Foster J. G., Mai W. F. (1973). A simplified medium for monoxenic culture of Pratylenchus penetrans and Ditylenchus dipsaci. Journal of Nematology.

[j_jofnem-2022-0052_ref_027] Santos J. C. F., Cunha A. J., Ferreira F. A., Santos R. H. S., Sakiyama N. S., Lima P. C. (2016). Herbaceous legumes intercropping in weed management of the coffee crop. Journal of Agriculture and Environmental Sciences.

[j_jofnem-2022-0052_ref_028] (2002-2003). SAS system for windows. Version 9.1.3.

[j_jofnem-2022-0052_ref_029] Silva R. A., Inomoto M. M. (2002). Host-range characterization of two Pratylenchus coffeae isolates from Brazil. Journal of Nematology.

[j_jofnem-2022-0052_ref_030] Souza V. H. M., Inomoto M. M. (2019). Host suitability of grain sorghum and sudangrass for Pratylenchus brachyurus. Arquivos do Instituto Biológico.

[j_jofnem-2022-0052_ref_031] Tomazini M. D., Silva R. A., Oliveira C. M. G., Gonçalves W., Ferraz L. C. C. B., Inomoto M. M. (2005). Resistência de genótipos de cafeeiros a Pratylenchus coffeae e Meloidogyne incognita. Nematologia Brasileira.

[j_jofnem-2022-0052_ref_032] Villain L., Salgado S. M. L., Trinh P., A. Sikora R., Coyne D., Hallmann J., Timper P. (2018). Plant parasitic nematodes in subtropical and tropical agriculture.

[j_jofnem-2022-0052_ref_033] Wilcken S. R. S., Mori E. S., Bacci M., Ferraz L. C. C. B., Oliveira C. M. G., Inomoto M. M. (2008). Relationships among Pratylenchus jaehni and P. coffeae populations from Brazil. Nematologia Brasileira.

[j_jofnem-2022-0052_ref_034] Wutke E. B., Calegari A., Wildner L. P., F. Lima Filho O., J. Ambrosano E., Rossi F., A. D. Carlos J. (2014). Adubação verde e plantas de cobertura no Brasil, vol. 1.

